# Production of Recombinant Human DNA Polymerase Delta in a *Bombyx mori* Bioreactor

**DOI:** 10.1371/journal.pone.0022224

**Published:** 2011-07-15

**Authors:** Yajing Zhou, Huiqing Chen, Xiao Li, Yujue Wang, Keping Chen, Sufang Zhang, Xiao Meng, Ernest Y. C. Lee, Marietta Y. W. T. Lee

**Affiliations:** 1 Institute of Life Sciences, Jiangsu University, Jiangsu, People's Republic of China; 2 Department of Biochemistry and Molecular Biology, New York Medical College, Valhalla, New York, United States of America; St. Georges University of London, United Kingdom

## Abstract

Eukaryotic DNA polymerase δ (pol δ) plays a crucial role in chromosomal DNA replication and various DNA repair processes. It is thought to consist of p125, p66 (p68), p50 and p12 subunits. However, rigorous isolation of mammalian pol δ from natural sources has usually yielded two-subunit preparations containing only p125 and p50 polypeptides. While recombinant pol δ isolated from infected insect cells have some problems of consistency in the quality of the preparations, and the yields are much lower. To address these deficiencies, we have constructed recombinant BmNPV baculoviruses using MultiBac system. This method makes the generation of recombinant forms of pol δ containing mutations in any one of the subunits or combinations thereof extremely facile. From about 350 infected larvae, we obtained as much as 4 mg of pol δ four-subunit complex. Highly purified enzyme behaved like the one of native form by rigorous characterization and comparison of its activities on poly(dA)/oligo(dT) template-primer and singly primed M13 DNA, and its homogeneity on FPLC gel filtration. In vitro base excision repair (BER) assays showed that pol δ plays a significant role in uracil-intiated BER and is more likely to mediate LP BER, while the trimer lacking p12 is more likely to mediate SN BER. It seems likely that loss of p12 modulates the rate of SN BER and LP BER during the repair process. Thus, this work provides a simple, fast, reliable and economic way for the large-scale production of human DNA polymerase δ with a high activity and purity, setting up a new platform for our further research on the biochemical properties of pol δ, its regulation and the integration of its functions, and how alterations in pol δ function could contribute to the etiology of human cancer or other diseases that can result from loss of genomic stability.

## Introduction

The complete and accurate replication of the genome is a central process in biology [1], [2]. In higher organisms this involves multiple processes as well as complex regulatory mechanisms that allow the temporally coordinated duplication of the chromosomes during the cell cycle. DNA replication is performed by a complex assembly of proteins, the replisome, at the heart of which are the DNA polymerases that drive the central process of DNA synthesis at the replication fork [3]. Among three major replicative polymerases, polymerase α (pol α), δ (pol δ), and ε (pol ε) in chromosomal DNA synthesis, pol δ plays a crucial role in DNA replication, and is adapted for the lagging strand synthesis [4], [5], but in addition also is a major participant in DNA repair processes [3], [6], [7].

Mammalian pol δ, discovered as a new eukaryotic DNA polymerase, consists of the p125 catalytic subunit which harbors both 5′-3′ DNA polymerase and 3′-5′-exonuclease activities and a tightly associated second subunit p50 [8]. This core is associated with two other subunits, p68 and p12, that are also referred to as the third and fourth subunit [9]–[11]. Thus, mammalian pol δ contains four subunits as a counterpart of fission yeast *S. pombe* pol δ which consists of four subunits, Pol3, Cdc1, Cdc27 and Cdm1 [12]–[14]. While in budding yeast, *S. cerevisiae* pol δ is a trimer of the first three subunits, Pol3p, Pol31p/Hys2, and Pol32p, encoded by two essential genes, *POL3* and *POL31*, and a non-essential gene *POL32*, respectively [15], [16]. It is surprising there have been comparatively few studies of this important enzyme in human/mammalian system, and our understanding of its fundamental biochemical properties lags behind those of replicative polymerases in other systems. Much of our progress has come from the studies in yeast system. One of the major obstacles is the availability of highly purified active pol δ either as a native form isolated from mammalian tissues or cultured cells and as a reconstitution form produced from other expression system. Performed in an early stage, the isolation of native pol δ from mammalian tissues was extremely tedious and only small amounts of enzyme protein could be isolated [17], [18]. Until 2002, a method was developed for the production of recombinant human pol δ and its subassemblies using baculovirus expression system in infected insect cells with his-tagged pol δ subunits or unmodified, *i.e.*, non-tagged subunits [19], [20]. This method involved co-infection with four separate baculoviruses which allowed for the reconstitution of the recombinant human pol δ enzyme. However, there were some problems of consistency in the quality of the preparations and the yields were still much lower than our expected. Since it depended on the co-infection of four different recombinant baculoviruses, there were variable amounts of subassemblies of pol δ present in the infected insect cell lysates, which meant additional purification and quality control experiments.

We have made a strong attempt to address this deficiency, and in this work, we present: a). construction of human pol δ by inserting the cDNAs for its four subunits into a single recombinant virus, each under the control of *polyhedrin* promoter in an individual expression cassette, using the baculovirus MultiBac system as described in [21]. This method makes the generation of recombinant forms of pol δ containing mutations in any one of the subunits or combinations thereof extremely easy. b). expression and its assembly of recombinant pol δ heterotetramer in *Bombyx mori* nucleopolyhedrovirus (BmNPV)–infected silkworm larvae using *Bombyx mori* as a bioreactor [22]. c). protein purification to near-homogeneity with a highly standardized protocol by home-made immunoaffinity column and FPLC chromatography on Mono Q column within 48 hours. As much as 4 mg of enzyme from about 350 infected larvae was obtained. d). characterization of purified enzyme by comparison of its specific activities, DNA elongation ability, and its homogeneity with the native form isolated from Hela cells on poly(dA)/oligo(dT) template-primer, singly primed M13 DNA, and FPLC chromatography on gel filtration column. Highly purified recombinant pol δ from BmNPV–infected silkworm larvae was shown to be functional similar to the native pol δ isolated from Hela cells. e). analysis of the role of pol δ involved in uracil-intiated BER *in vitro*. It seems likely that loss of p12 modulates the rate of SN (single-nucleotide) BER and LP (long patch) BER during the repair process.

Our researches provide a simple, fast, reliable and economic way for the large-scale production of human DNA polymerase δ with a high specific activity and high purity. Thus, this work sets up a new platform for our current research on the biochemical properties of pol δ, its regulation and the integration of its functions, and how alterations in pol δ function could contribute to the etiology of human cancer or other diseases that can result from loss of genomic stability.

## Materials and Methods

### Reagents and chemicals

All reagents and chemicals used in this study were purchased from Sigma-Aldrich, Gibco-BRL, and Invitrogen except as otherwise indicated. Mouse polyclonal antibody against p50 (ZJM5002), rabbit polyclonal antibodies against p125 (ZJR12501), p68 (ZJR6803) and p12 (ZJR1204) were prepared in our laboratory, respectively.

### Construction of recombinant baculoviruses containing multigene expression cassettes

**Figure 1:**
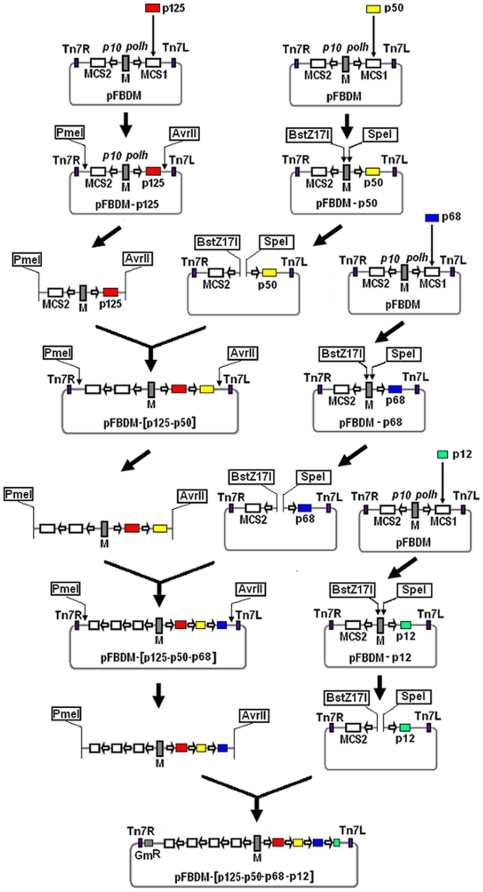
A strategy for assembling multigene expression cassettes in pFBDM. Four genes, p125, p50, p68, and p12, were inserted into MCS1 between *Bam*HI and *Xba*I sites, respectively. The expression cassette containing p125 was excised by digestion with *Avr*II and *Pme*I (boxed) and placed into a multiplication module (M) of a construct containing p50 via *Bst*Z17I/*Spe*I sites. Followed the same logic, a pFBDM-[p125-p50-p68] was generated by an insertion of the expression cassette containing p125 and p50 into a construct containing p68. Finally, a recombinant transfer vector pFBDM-[p125-p50-p68-p12] was generated in which each subunit is under the control of individual *polyhedrin* promoter.

Four cDNA fragments coding the full-length sequence of pol δ individual subunit were excised between the *Bam*HI and *Xba*I sites from the vector pCDNA3.1(+)-FLAG [23] containing the *Bam*HI/*Eco*RI insertion of p125, p50, p68, or p12, respectively [24]. To construct the recombinant transfer vector pFBDM-[p125-p50-p68-p12] for multigene expression, the logic of adding subunits into polycistronic vector pFBDM is illustrated in Figure 1 with MultiBac system, according to manufacturer's instruction (kindly provided by Dr. Timothy J. Richmond, Institute for Molecular Biology and Biophysics, ETH Zürich, Switzerland) [21].

The obtained recombinant plasmid pFBDM-[p125-p50-p68-p12] was then transformed into *E. coli* BmDH10Bac competent cells which contain the BmNPV bacmid with a miniF replicon, kanamycin-resistant gene, *lac*Z gene, mini-attachment Tn7 site, and a helper plasmid [25], [26]. Recombinant bacmid was obtained through the transposition of the Tn7 elements from the pFBDM derivative to the mini-attachment Tn7 target site on the BmNPV bacmid. High molecular weight mini-prep DNA was prepared from a selected white phenotype *E. coli* and used to transfect silkworm BmN cells for the production of recombinant baculovirus particles, according to manufacturer's instruction (Invitrogen).

### Expression and purification of recombinant human DNA pol δ

The variety of silkworm used in this work was “306” held by our laboratory. About 350 larvae were used as the bioreactors for the production of human polymerase delta. The second day of fifth instar larvae were injected with 10 µl of viral solution (3.1×10^7^ pfu/ml) per *os*. After 5 days post injection, infected larvae were weighed, and the hemolymph was collected and frozen at −80°C.

All purification steps were carried out at 4°C. The immunoaffinity column, used for pol δ complex purification, coupled with a polyclonal antibody (ZJR12501) against human catalytic subunit p125, was prepared using CarboLink™ Immobilization Kit according to manufacturer's instruction (Pierce). Collected larval hemolymph was centrifuged at 12,000 rpm for 30 min and divided into two fractions. The supernatant fraction was diluted three times with TGEE buffer (40 mM Tris-HCl, pH 7.8, 10% glycerol, 0.5 mM EDTA, 0.1 mM EGTA) containing 0.1 M NaCl, 1 mM phenylmethylsulfonate, and protease inhibitor mixture, and then subjected to immunoaffinity chromatography (20 ml agarose beads) and FPLC Mono Q 5/50 GL column (GE Healthcare) as previously described [24]. Eluted fractions were analyzed by 12.5% SDS-PAGE and Coomassie Blue staining.

In another protocol, the pellet fraction containing cell debris and tissues was resuspended in lysis buffer (20 mM Tris-HCl, pH 7.8, 10% glycerol, 0.5 mM EGTA, 1 mM EDTA, 1 mM MgCl_2_, 200 mM NaCl, 0.1% NP-40, 1 mM phenylmethylsulfonate, and protease inhibitor mixture) on ice for 30 min, disrupted by sonicating using 4×15 second bursts with a 15 second cooling period between each burst, and centrifuged at 14,000 rpm for 60 min. Collected supernatant was diluted three times with TGEE buffer and purified further on immunoaffinity column and FPLC Mono Q column using the same protocol used for purification of supernatant fraction of hemolymph.

### FPLC gel filtration chromatography

A 250 µl sample from the peak fraction after FPLC Mono Q ion exchange chromatography was loaded on a Superdex 200 column equilibrated with 150 mM NaCl in TGEED buffer (TGEE plus 1 mM dithiothreitol). The column was eluted at a flow rate of 0.25 ml/min, and a total of 90 fractions of 0.25 ml each were collected.

### Purification of the pol δ complex from Hela cells

A protocol used for purification of pol δ complex from Hela cells was essentially as described for its isolation from HEK 293T or Hela cells [27], [28]. Total lysates from 80 plates (about 1.5×10^8^ cells) were loaded onto a 10-ml immunoaffinity column. Eluted fractions across the peak were separated by 12.5% SDS-PAGE and stained with Coomassie Blue or transferred to nitrocellulose membrane for western blot analysis.

### Purification of PCNA

Recombinant PCNA was expressed in *E. coli* and purified to near homogeneity by a protocol as previously described [29], and made some modifications. One liter of DH5-α cells harboring human PCNA in pTACTAC vector were induced with 0.3 mM IPTG for 18 hours at 27°C. The cells were harvested by centrifugation at 4,000 rpm for 30 min, resuspended in 200 ml of lysis buffer (25 mM Tris-HCl, 1 mM EDTA, 25 mM NaCl, 0.01% Nonidet P-40, 2 mM benzamidine, 2 mM pepstatin A, 1 mM PMSF, 1 mM DTT, 100 ug/ml lysozyme, pH 7.4), and disrupted by sonicating. The lysates were centrifuged at 14,000 rpm for 60 min at 4°C. The supernatant was loaded onto a Q-sepharose column (2.5×38 cm) equilibrated with TGEED buffer. The column was washed with 2 bed volumes of TGEED containing 0.2 M KCl, followed by a linear gradient of KCl from 0.2 to 0.8 M (1,800 ml) at 2 ml/min. 8-ml fractions were collected and PCNA was eluted between 0.4 and 0.5 M salt. Peak fractions were pooled and dialyzed against TGEED. The dialyzed material was then loaded onto a 4-ml Mono-P HR 5/20 column equilibrated with TGEED. The column was washed with 2-bed volumes of TGEED followed by 80 ml of a 0.2–0.8 M KCl gradient at 1 ml/min. PCNA was eluted around 0.4–0.5 M KCl.

### DNA polymerase assay

Assays for DNA polymerase activity were performed by determination of [^3^H]dTMP incorporated into poly(dA)/oligo(dT) template/primer in the presence and absence of PCNA as described previously [24], [27]. One unit of DNA polymerase activity corresponds to the incorporation of 1 nmole of dTMP per hour at 37°C. Assays using singly primed M13 DNA as the template were performed as previously described [24]. The reactions were started by incubation at 37°C for 30 min and stopped by addition of 20 mM EDTA. 5 µl aliquots of each reaction were spotted onto DE81 papers which were washed 3 times with 0.3 M ammonium formate pH 7.8, once with 95% ethanol, dried and counted using liquid scintillation counter. The rest of the products were run on 1.5% alkaline agarose gel at 50 V for 2.5 hours. The gel was dried and the product length was visualized with a phosphoImager or evaluated on an x-ray film.

### DNA ladder and oligonucleotide labeling

A 1 kb DNA ladder (New England Biolabs) for M13 assays and 25-mer oligonucleotide (5′-GCCACTACAGCACCTTGACAGCCAG-3′) [28] as a DNA maker for BER assays were end-labeled with [γ-^32^P]ATP (5000 Ci/mmol, MP Biochemicals) in 1×T4 kinase buffer containing T4 polynucleotide kinase (New England Biolabs), followed by purification using the QIAquick nucleotide removal kit (Qiagen).

### Cellular extracts for In vitro BER assay

The wild-type mouse embryonic fibroblast (MEF) cell line and the matched littermate pol β-knockout cell line [30] were the generous gifts from Dr. Samuel H. Wilson. The conditions for cell culture and preparation of cell extracts have been described previously [31], [32].

### In vitro BER assay

A uracil-containing plasmid pUC19N, derived from pUC19 with additional 48-mer *N.Bst*NB1 fragment, designed to discriminate total BER, SN BER, and LP BER, was a generous gift provided by Dr. Samuel H. Wilson and described as previously [32], [33]. Upon digestion with restriction enzyme *Kpn*I, it would generate a 41-bp fragment representing total BER product, while digestion with *Xho*I and *Kpn*I, it would generate a 25-bp fragment representing SN BER plus LP BER and a 16-bp fragment representing LP BER. For BER assays with cell extract, 10 µg of MEF extract prepared from wild-type or pol β −/− cells were analyzed in a final volume of 10 µl containing 10 nM plasmid DNA in 50 mM Tris-HCl (pH 7.5), 5 mM MgCl_2_, 20 mM NaCl, 1 mM DTT, 4 mM ATP, 20 µM each dATP, dGTP, and dTTP, and 2.3 µM [α-^32^P]dCTP. In experiments for depleting pol δ, the antibodies (ZJR12501, ZJM5002, ZJR6803, and ZJR1204) specific for pol δ four subunits were incubated with the cell extract by rotation for 60 min at 4°C; protein A/G agarose beads were then added; the mixture was incubated for another 30 min, and the beads were removed by centrifugation at 2500 rpm for 5 min, before the repair reaction. The BER reaction mixture was incubated at 37°C for 30 min. After phenol/chloroform extraction and ethanol precipitation, the recovered reaction product was subjected to the restriction-enzyme analysis. The digested products were separated by electrophoresis on 16% denaturing polyacrylamide gel containing 8 M urea. Radioactive products were visualized by phosphorimaging and analyzed with ImageQuant software (Amersham Biosciences, NJ). For the calculation of SN BER, arbitrary phosphorimager units in the 16-bp fragment were subtracted from that of 25-bp fragment. For reconstitution BER assay with purified recombinant proteins, the assays were performed under similar reaction conditions as above except that the cell extract was replaced by the following proteins as indicated: I unit of *E. Coli* UDG (New England Biolabs), 2.5 units of recombinant human APE-1(New England Biolabs), 1 unit of T4 ligase (Promega), 2 unit of recombinant human FEN-1 (Proteome Resources, LLC), 100 ng of recombinant human PCNA, and 100 fmol of recombinant human pol δ complexes.

### Western blot analysis

Western blotting was performed as previously described [20]. Antibodies used in this experiment were ZJM5002, a mouse polyclonal antibody against p50, ZJR12501, ZJR6803 and ZJR1204 which are rabbit polyclonal antibodies against p125, p68 and p12, respectively. After electrophoretic transfer, the nitrocellulose membrane bounded by target proteins was stained with Ponceau S and cut into four pieces according to the molecular weight of each subunit. Four pieces of membrane were blocked with 5% w/v nonfat dry milk in TBST buffer (20 mM TrisHCl, pH 7.4, 150 mM NaCl, 0.05% Tween 20) for 1 hour at room temperature. The blot was then incubated with individual primary antibody corresponding to each subunit for 1 hour at room temperature or overnight at 4°C. After three 15-min washes with TBST buffer, the blot was incubated with AP-conjugated or HRP-conjugated goat anti-mouse IgG or goat anti-rabbit IgG for 1 hour and washed with TBST buffer 3 times for 10 min. Perfect Protein Western Blot Kit (Novagen) was used for signal generation.

### Protein determination

Protein was determined by the Bradford method with bovine serum albumin as a standard, or by “in-gel” analysis of protein concentration of the catalytic subunit p125 using catalase as a protein standard [28].

## Results and Discussion

### Construction of recombinant transfer vector containing pol δ four-subunit gene expression cassettes

The earlier study of the enzymology of the mammalian DNA pol δ had been extraordinarily difficult due to the small amounts of enzyme protein available [10], [17], [18]. Rigorous purification of the enzyme culminated in an isolation of two-subunit enzyme containing a catalytic subunit of 125 kDa and a second subunit of 50 kDa [17], [34]. In previous studies [20], [24], we had developed a method to reconstitute the heterotetrameric form of pol δ by co-expression of four separate recombinant baculoviruses for its individual subunit in Sf-9 insect cells. Since this method depended on the co-infection of four different viruses, there existed variable amounts of subassemblies of pol δ in infected cell lysates, resulting in the problems of consistency in the quality of the preparations.

In order to address this deficiency, in this work, a construction of recombinant baculovirus containing multigene expression cassettes was made using the MultiBac system as described [21]. Here, we chose to use the unmodified subunits, rather than any tagged proteins that could result in the assembly of complexes which might have modified or compromised properties. The detailed logic of adding subunits is shown in Figure 1 in which a recombinant transfer vector pFBDM-[p125-p50-p68-p12] containing four gene expression cassettes was generated. Due to the multiplication module between the two promoters, the pFBDM is particularly suited for generating multigene expression cassettes, making the generation of recombinant forms of pol δ containing mutations in any one of the subunits or different subunit combinations thereof extremely easy.

Our previous studies indicated that the fourth subunit p12 was not a passive structural element for the stability of pol δ, but that its interaction with the core dimer produced alterations in the conformation and/or structure of the catalytic site that are essential for the catalytic properties of the pol δ holoenzyme [24], [28]. Pol δ itself may be a target of the DNA damage response. As genotoxic agents and replication stress triggered the degradation of the p12 subunit, pol δ was converted to a trimeric form, whose altered responses to encounters with DNA lesions might contribute to the DNA damage response [28]. In order to provide a foundation for assessing the contributions of p12 and p68 on the functions of the catalytic core enzyme consisting of p125 and p50, a rigorous analysis of the enzymatic behavior of pol δ and its subassemblies will be needed. Therefore, by the use of a similar strategy, we also constructed the recombinant transfer vectors, pFBDM-[p125-p50] for pol δ core, two trimers, pFBDM-[p125-p50-p12] and pFBDM-[p125-p50-p68] lacking p68 or p12, respectively.

### Generation of recombinant BmNPV baculovirus

**Figure 2:**
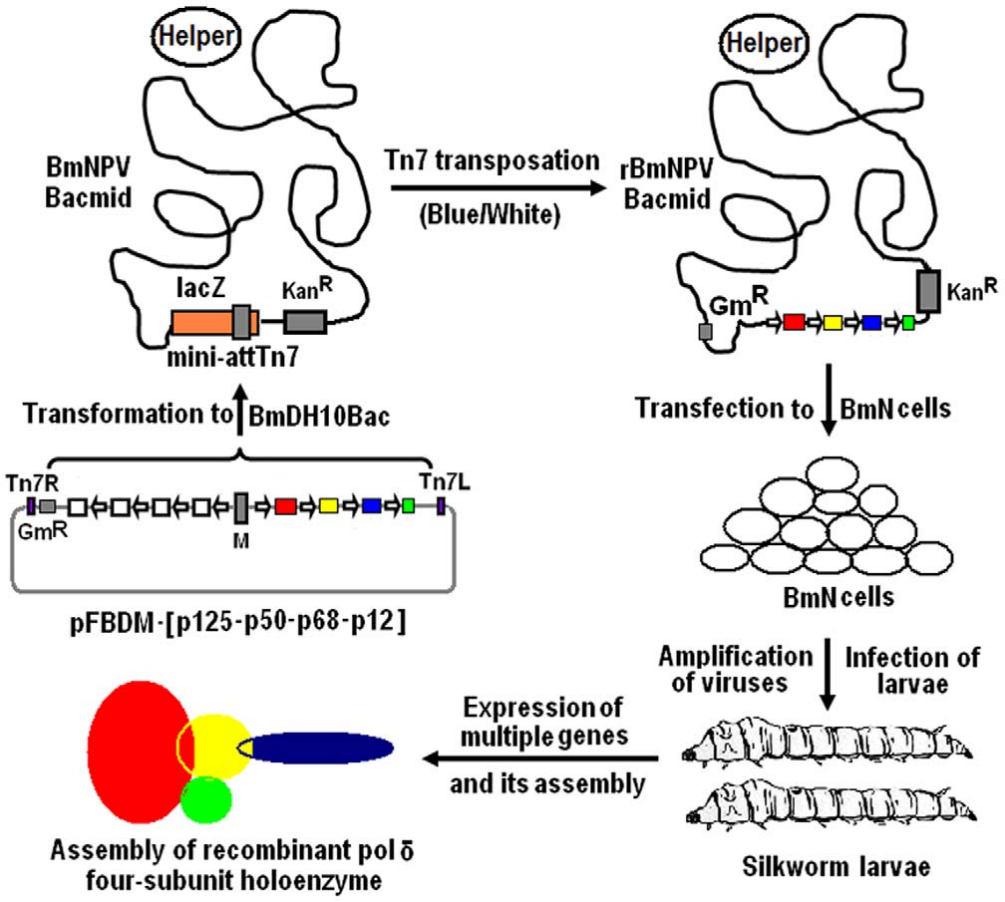
A schematic outline for generation of recombinant virus and its expression in silkworm larvae. The obtained recombinant transfer vector pFBDM-[p125-p50-p68-p12] was introduced into BmNPV bacmid DNA in BmDH10Bac *E. coli* cells. Colonies containing bacmid carrying integrated four-gene expression cassettes were identified by blue/white screening. Bacmid DNAs were isolated from selected white phenotypes. The recombinant BmNPV viruses were generated by the transfection of BmN insect cells with isolated bacmid DNAs. The four subunit pol δ complex was then expressed and assembled in silkworm larvae by the infection of larvae with prepared recombinant viruses.

**Figure 3:**
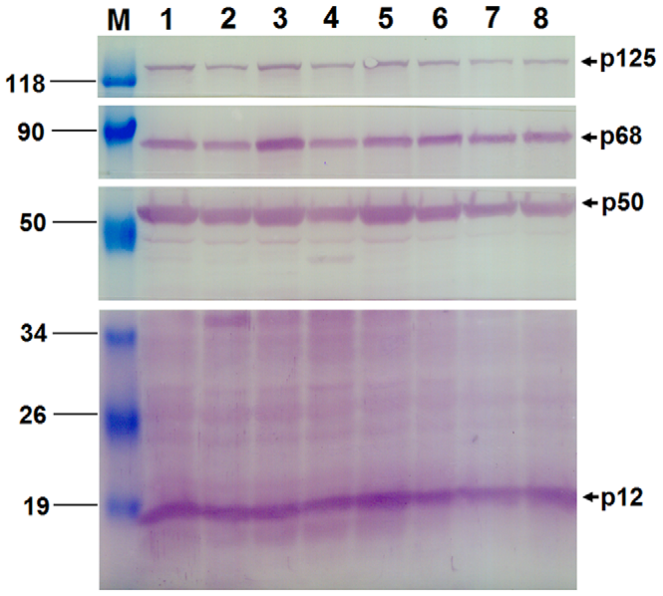
Western blotting analysis for the generation of the recombinant BmNPV viruses in BmN cells. Eight bacmid DNAs, isolated from the white clones and verified by PCR analysis, were transfected into BmN cells. After 5 days post-transfection, each supernatant was harvested as the viral stocks and the cell pellets were analyzed by Western blotting using the antibodies specific for the individual subunit as described under “Materials and Methods”. M indicates the positions in kDa of protein markers. The lanes from 1 to 8 show eight independent transfections of BmN cells with recombinant BmNPV bacmid DNAs. Pol δ four subunits are marked by arrows.

Since Maeda et al. first reported the production of human α-interferon in silkworm using recombinant BmNPV [35], silkworm larvae have been used as a bioreactor for the production of huge recombinant proteins for decades. Recently a novel Bac-to-Bac system using BmNPV is established by the construction of BmNPV bacmid DNA with a miniF replicon (*Escherichia coli* replication), kanamycin-resistant gene, *lac*Z gene, mini-attachment Tn7 site [25], which possesses a comparable time saving, high performance and high efficiency to Bac-to-Bac baculovirus expression system using AcMNPV distributed from Invitrogen Corp. In this work, we constructed a recombinant BmNPV baculovirus for expression of pol δ heterotetramer using this novel system. A schematic outline for generation of recombinant virus is shown in Figure 2. The obtained donor plasmid pFBDM-[p125-p50-p68-p12] was transformed into *E. coli* BmDH10Bac competent cells. The white clones were selected and BmNPV bacmid DNAs were isolated. Eight correct phenotypes verified by PCR analysis were selected for the transfection of BmN cells. After 5 days post-transfection, the recombinant baculoviruses containing four subunit gene expression cassettes were successfully obtained based on the Western blotting analysis as shown in Figure 3. In addition, we also prepared a set of recombinant viruses for pol δ subassemblies: dimer enzyme pol δ-core, trimer pol δ-p12 lacking p12 subunit and trimer pol δ-p68 lacking p68, respectively. The transfections of BmN cells were identified by Western blotting analysis as shown in Figure S1.

### Expression and purification of the recombinant human pol δ four-subunit enzyme in silkworm larvae

The silkworm, *Bombyx mori*, has been used for silk production for centuries. Recently, as an important well-identified model organism in genetics, physiology, and biochemistry, it has been also expended as a bioreactor for the production of recombinant proteins not only for research applications but also for commercial purposes. In general, the expression levels of recombinant proteins in silkworm larvae are higher than those in cultured insect or mammalian cells. The activities of human butyrylcholinesterase from the hemolymph of infected silkworms were 23 or 280 times higher than those in infected BmN or CHO cells [36]. From the hemolymph of ten silkworm larvae, about 1 mg of human macrophage colony-stimulating factor (M-CSF) could be isolated [37]. Even from 1 ml of collected hemolymph, 0.16 mg of human growth factor could be obtained by purification [38]. While we have developed efficient methods for the expression and purification of pol δ and its subassemblies, we have sought to improve both the quality and yields to facilitate our work. Here we tested the expression and assembling of human pol δ four-subunit complex in silkworm larvae for the first time. The silkworm “306”, a BmNPV-sensitive variety, was chosen as a bioreactor. After infection of the fifth instar larvae by the injection with recombinant baculovirus for four-subunit expression, about 50 ml of hemolymph from 350 infected larvae was collected and the expression levels were judged by Western blotting (data not shown).

**Figure 4:**
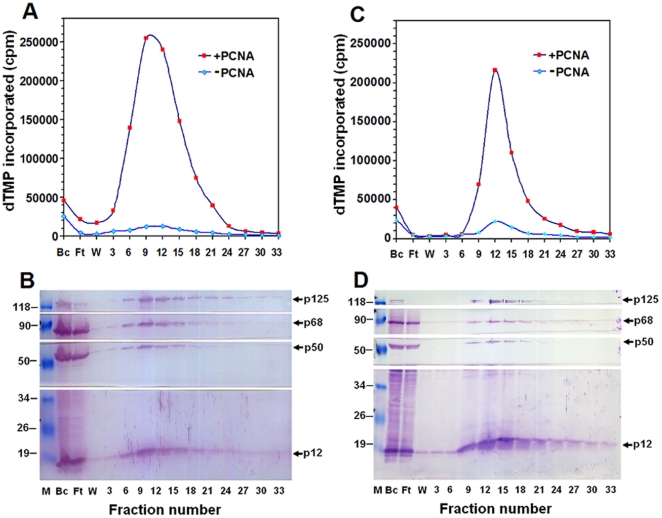
Purification of recombinant human pol δ heterotetramer by immunoaffinity chromatography. Infected larval hemolymph was centrifuged and divided into supernatant and pellet fractions. The supernatant fraction was subjected to immunoaffinity column. The lysates (BC), flow-through (FT), wash (W), and eluted fractions were assayed for DNA polymerase activity in the presence (square) and absence (diamond) of PCNA (panel A), and analyzed by 12.5% SDS-PAGE followed by Western blotting (panel B) using the antibodies specific for the individual subunit as described under “Materials and Methods”. A parallel purification for the pellet fraction was also performed. The activities were assayed (panel C) and Western blotting analysis was carried out (panel D).

To facilitate the purification of the intact complex, we developed a highly standardized protocol by rapid isolation of recombinant pol δ heterotetramer through immunoaffinity chromatography and FPLC chromatography on Mono Q column within 48 hours and storage at high protein concentration in liquid nitrogen. This procedure allowed the purification of the pol δ four-subunit complex in an intact form to near-homogeneity and kept the activity stable for at least 2 years. The purification was monitored by both pol δ activity assays and Western blotting analysis. When the diluted supernatant faction after centrifugation of collected hemolymph was loaded on the immunoaffinity column, the pol δ activities were eluted between the fraction 6 and 18. The activities around peak were stimulated by PCNA near 20-fold (Figure 4A). The fractions across peak contained all four subunits of the expected molecular masses as judged by Western blotting analysis (Figure 4B). In this step, we applied a “column overloading” on immunoaffinity chromatography, assuring a minimal non-specific protein binding. Therefore, there was still a small amount of p125 in the flow-through fraction (Figure 4B). In another protocol, when the supernatant from the lysate of pellet fraction of hemolymph went through immunoaffinity column, the activities were eluted between faction 9 and 18 and the peak activity was stimulated by PCNA about 10-fold (Figure 4C). Similar to the behavior of supernatant faction of hemolymph on the column, the fractions across peak also contained all four subunits of the expected molecular masses (Figure 4D). In later case, we loaded a low protein concentration of the sample onto the column which is below the binding capacity of immunoaffinity beads. There was no any extra p125 in the flow-through fraction where all p125 assemblies are bound onto the beads (Figure 4D). However, in both cases, a comparable amount of other three subunits in the flow-through fraction indicates a possibility of that a subassembling occurred besides the four-subunit complex during the expression of pol δ in infected silkworm larvae.

**Figure 5:**
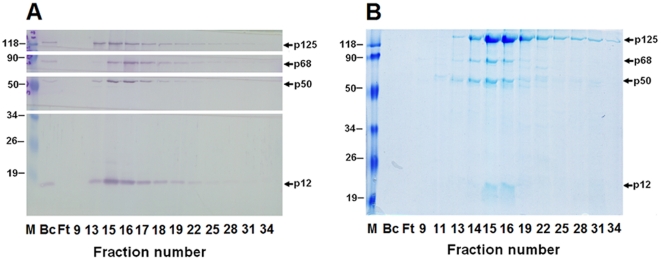
Purification of recombinant pol δ heterotetramer by Mono Q chromatography from supernatant fraction of hemolymph. The peak fractions (fractions 6–21) from the immunoaffinity chromatography step in which the column was applied with supernatant fraction of hemolymph were combined and passed through a Mono Q column. The lysates (BC), flow-through (FT), and eluted fractions were analyzed by 12.5% SDS-PAGE followed by Western blotting (panel A) or Coomassie Blue staining (panel B).

The eluted fractions between 6 and 21 from immunoaffinity column applied with supernatant faction of hemolymph (Figure 4A and Figure 4B) were combined, dialyzed, and loaded on a Mono Q 5/50 GL column. The peak activities of pol δ were eluted between 350 and 400 mM NaCl in TGEED buffer (data not shown). Eluted fractions were judged by 12.5% SDS-PAGE and stained with Coomassie Blue (Figure 5B) or Western blotting analysis (Figure 5A). Thus, a protein complex containing four subunits was obtained as expected molecular masses. From 350 larvae, we yielded about 4 mg of four-subunit protein complex from the pooled Mono Q peak fractions 14–17 purified to near homogeneity by our highly standardized protocol. A summary for the specific activities and yield of preparations for purification of recombinant pol δ holoenzyme was shown in table 1.

**Table 1:** Purification of recombinant DNA polymerase δ from infected silkworm larvae^a^.

*Step*	*Total volume (ml)*	*Protein (mg)*	*Activity (Units)*	*Specific activity (Units/mg)*
Supernatant of hemolymph	50	297.5	ND^b^	—
Immunoaffinity	112.5	10.33	115696	11200
Mono Q	2.0	4.01	100250	25000

a The purification procedure was carried out as described under Materials and Methods. DNA polymerase activity was followed by the assays on poly(dA)/oligo(dT) template-primer.

b Activity in supernatant of hemolymph cannot be accurately measured.

Isolation of multi-subunit pol δ from mammalian tissues has been extremely difficulties. One of them is the case of the p68 subunit which is prone to proteolytic nicking [9], [11]. Rigorous isolation of natural mammalian pol δ resulted in no detectable p66 subunit in protein preparations [17], [18], [34]. Even in the protocol which demonstrated co-purification of p66 with p125 and p50, the p66 subunit was subject to proteolytic degradation and stoichiometrically under-represented in the purified preparation [11]. In this study, the four-subunit human pol δ complex was reconstituted by overexpression in silkworm larvae with the recombinant BmNPV containing four subunit gene expression cassettes. Rigorous isolation of pol δ activity from infected larvae led to the isolation of a near homogeneous pol δ heterotetramer in an intact form. No degradation of the p68 subunit was observed in our preparation (Figure 5).

Recent studies discovered a novel cellular response to DNA damage. Exposure of mammalian cells to UV light or alkylating agents led to the rapid degradation of the p12 subunit. Pol δ was consequently converted from a heterotetramer *in vivo* to a trimer lacking the p12 subunit [27]. The loss of the p12 did not result in the dissociation of the remaining subunits. This converted trimer *in vivo* could be isolated as an intact complex of p125, p50, and p68 using immunoaffinity chromatography with altered properties and behaviors when it encountered DNA base lesions [27], [28]. Previous studies also indicated that highly purified pol δ enzymes by immunoaffinity chromatography were polydisperse on gel filtration. Further examination of the size of pol δ by native gel electrophoresis gave results which indicated the existence of discrete complexes [9]. There are major questions raised as to whether there exist subassemblies *in vivo* due to its intrinsic property of pol δ. In this work, when the sample was applied with the pellet fraction of hemolymph containing cell debris and tissues, the pol δ subassemblies were separated and appeared in Mono Q fractions (Figure S2). There are predominant phenotypes of catalytic subunit p125 alone in fraction 12, dimer pol δ-core (p125/p50) in fraction 15, trimer pol δ-p12 lacking p12 in fraction 16, and four-subunit pol δ complex in fraction 17, respectively. This result implies the hypothesis that pol δ might be a mixture of subassemblies *in vivo* and contributes to the cellular response to different events as a component, one of pol δ subassemblies, of the multi-protein replication and DNA repair complexes.

### Characterization of isolated recombinant four-subunit human DNA pol δ from supernatant fraction of hemolymph of infected silkworm larvae

**Figure 6:**
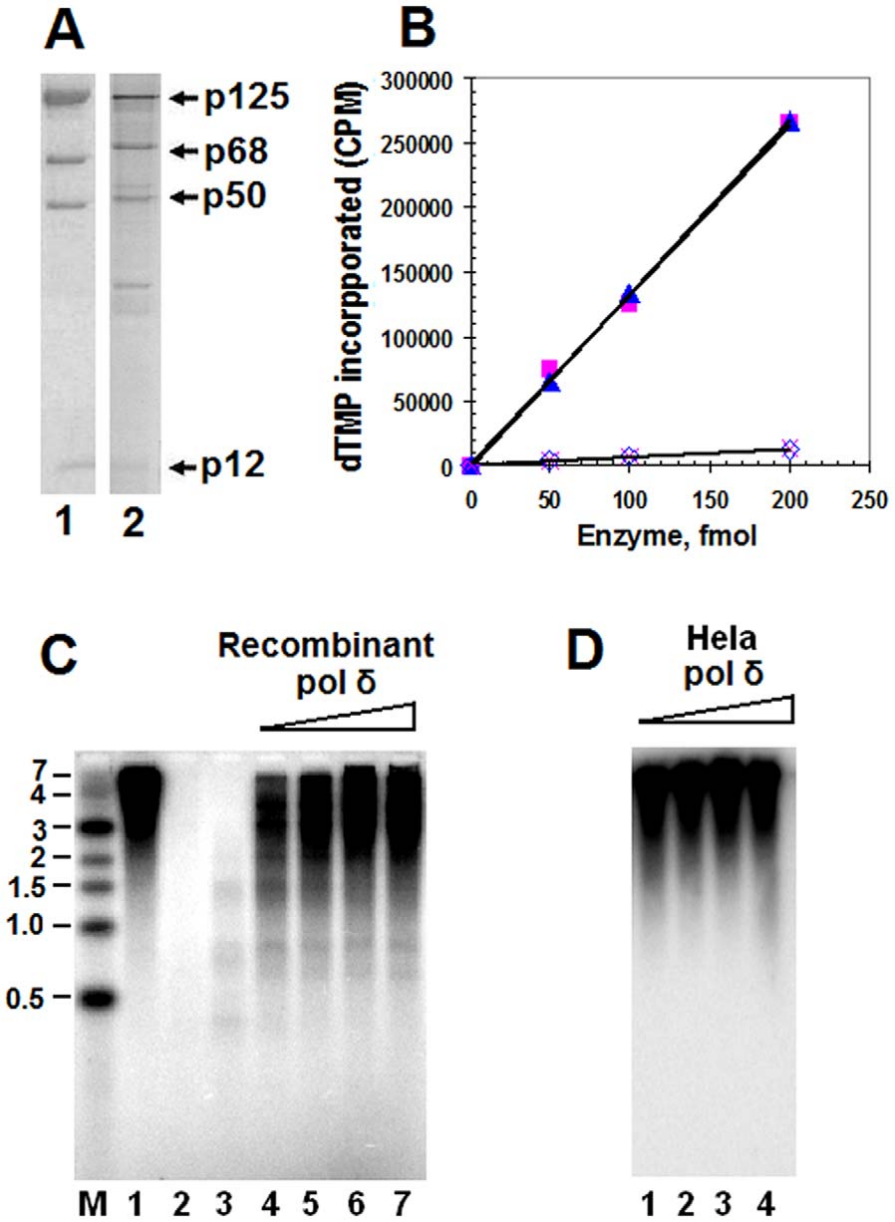
Characterization of recombinant pol δ heterotetramer complex. Panel A: Coomassie Blue protein stain of the pol δ enzymes used in this experiment. Lane 1 shows the recombinant pol δ enzyme isolated from supernatant fraction of infected silkworm larvae hemolymph after Mono Q chromatography step. Lane 2 shows the native pol δ enzyme isolated from cultured Hela cells by immunoaffinity chromatography. Panel B: A direct side-by-side comparison of recombinant pol δ enzyme with the most highly purified native pol δ enzyme from Hela cells on its specific activities and response to PCNA (▪, native pol δ with PCNA; ▴, recombinant pol δ with PCNA; ⋄, native pol δ without PCNA; ×, recombinant pol δ without PCNA). Panel C: Analysis of DNA products synthesized by recombinant pol δ holoenzyme on primed M13 DNA as described under “Materials and Methods”. Horizontal bars (Lane M) on the left indicate the positions in kb of DNA markers. Lane 1–2 show 200 fmoles of native pol δ isolated from Hela cells as the positive controls in the presence (Lane 1) and absence (Lane 2) of PCNA. The Lanes from Lane 3 to 7 show increasing amounts of recombinant pol δ enzyme (fmole) containing 0, 50, 100, 150, and 200, respectively. Panel D: Equal amounts of native form pol δ enzyme from Hela cell preparation analyzed in holoenzyme assay. The lanes from left to right show the increasing amounts of native enzyme of 50, 100, 150, 200 fmoles.

The specific activity of the recombinant pol δ isolated by Mono Q chromatography from the supernatant fraction of hemolymph (Figure 6A1) was compared with that of native pol δ isolated from Hela cells by immunoaffinity chromatography (Figure 6A2) on sparsely primed poly(dA)/oligo(dT) template-primer in the presence and absence of PCNA. By the definition of one unit as an incorporation of 1 nmole of dTMP per hour, the specific activity of isolated recombinant pol δ enzyme was calculated to be 25,000 units/mg, almost the same as the 25,600 units/mg of purified native form pol δ from Hela cells in this work (Figure 6B). This value is also comparable to the 9,000 units/mg and 26,400 units/mg of characterized pol δ preparations isolated from calf thymus [11], [18]. The stimulation level of isolated recombinant pol δ by PCNA was about 20 times, with the same stimulation level as our preparation from Hela cells and comparable to the one (16-fold) isolated from calf thymus [18].

To test more directly DNA elongation activity of our isolated recombinant pol δ from Mono Q step, we analyzed its activity in the holoenzyme assay in which pol δ activity is dependent on the presence of RFC for the loading of PCNA. As expected, it efficiently elongated primer DNA on single strand M13 DNA template. The products generated in holoenzyme assay were analyzed by electrophoresis on 1.5% alkaline-agarose gel followed by autoradiography. By a 30-min incubation in the presence of auxiliary factors, 100 fmoles of recombinant pol δ efficiently replicated singly primed M13 DNA mostly to completion, near 7 kb (Figure 6C). Its behavior is similar to the native form pol δ from Hela cell preparation (Figure 6D). Thus, our preparation of recombinant pol δ isolated from the supernatant fraction of infected larvae hemolymph is functionally indistinguishable from the native enzyme prepared from either our preparation from Hela cells or calf thymus by immunoaffinity chromatograph [11], [18].

**Figure 7:**
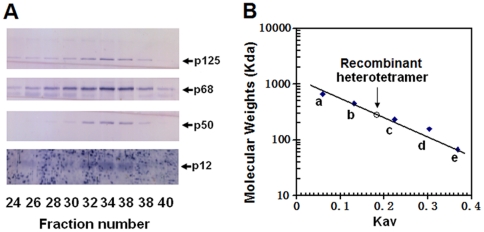
Molecular weight determination of recombinant pol δ heterotetramer complex. A 250 µl of sample from Mono Q peek fraction 15 was passed through a Superdex 200 column precalibrated with molecular weight standards. Panel A: The fractions across the peak of polymerase activity were run on 12.5% SDS-PAGE and Western-blotted with the indicated antibodies. The pol δ four subunits are marked by arrows. Panel B: The diagram shows the calibration of the column that was used to estimate the molecular weight. The standards used here were: a, thyroglobulin (667,000); b, ferritin (445,000); c, catalase (232,000); d, aldolase (158,000); e, bovine serum albumin (67,000). The elution position of the recombinant heterotetramer complex is indicated by a vertical arrow. A calibration curve was plotted by the logarithm of molecular weights versus calculated Kav.

There was a contrast of molecular weights of pol δ complexes between the yeast and mammalian system. Previous studies reported a dimeric structure-like molecular weight of yeast pol δ complexes both in *S. cerevisiae* and in *S. pombe* [13], [15], [16]. However, a more careful reinvestigation indicated that the yeast pol δ complex was not dimeric and the initial findings were attributed to the result of an anomalous migration of the pol δ complex on gel filtration due to an asymmetric structure [39]. In the case of mammalian pol δ, a 280,000 of molecular weight of a heterotetrameric pol δ complex, reconstituted in infected Sf-9 cells, was reported by comparison to protein standards on Superose 6 gel filtration [20]. A more accurate molecular weight of 230,000 was determined by a calculation from the Stokes radii and the sedimentation coefficients. To examine the physical properties of recombinant human pol δ complex isolated from infected silkworm larvae hemolymph, its molecular weight was estimated by comparison to protein standards on Superdex 200 gel filtration using a logarithm plot of its molecular weight versus elution volume. A 250 µl of sample from Mono Q peek fraction 15 (Figure 5B) was passed through a Superdex 200 column. Western blotting analysis of the collected fractions showed that all four subunits were co-eluted around peak fraction 34 and there was no apparent loss of subunits during this step, indicating that it did not behave anomalously (Figure 7A). The estimated molecular weight was about 280,000 based on a calibration curve as shown in Figure 7B, which is consistent with those previously reported for the native pol δ complex and the reconstituted form from the infected Sf-9 cells [9], [20].

### Pol δ plays a significant role in base excision repair

**Figure 8:**
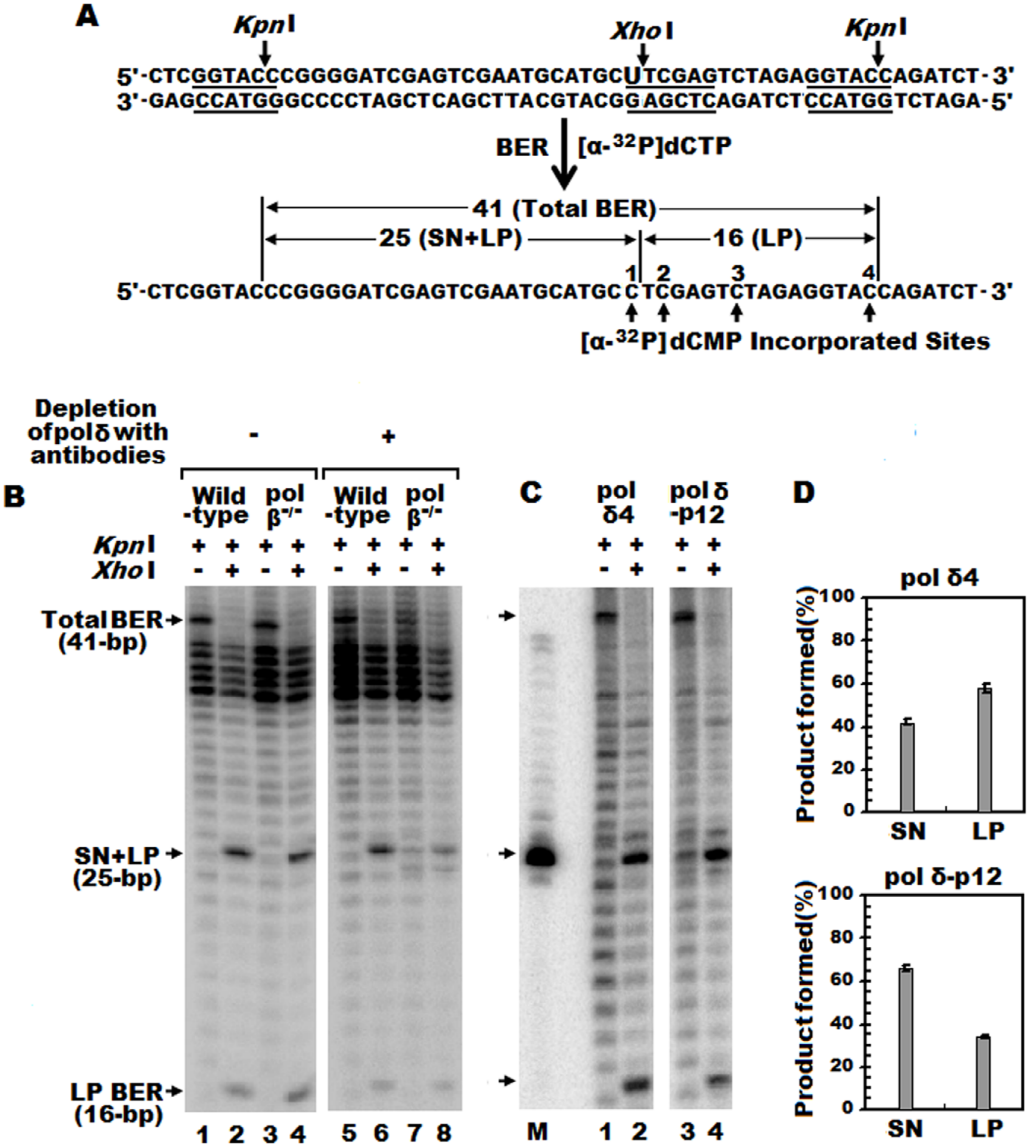
Contribution of pol δ to SN and LP BER of uracil-containing plasmid DNA. Panel A: A schematic representation of the uracil-containing plasmid DNA with *Kpn*I and *Xho*I restriction-enzyme sites (underlined). After BER reaction, the repaired products were restricted with indicated enzymes, and digested DNA fragments were separated on 16% denaturing polyacrylamide gel containing 8 M urea. DNA fragment sizes and descriptions of DNA synthesis products are indicated. Panel B and C: Autoradiograph of gel electrophoresis to measure *in vitro* BER products. The BER reaction was performed with cellular extracts (B), from wild-type MEF (lane 1, 2), pol β −/− (lane 3, 4) cells, pol δ–depleted wild-type MEF (lane 5, 6), and pol δ–depleted pol β −/− (lane 7, 8). The BER reaction was performed with purified enzymes (C), pol δ4 (lane 1, 2) and pol δ3-p12 (lane 3, 4). Lane M indicates a ^32^P labeled 25-bp oligonucleotide as a DNA maker. Panel D: The ratio of SN BER and LP BER with purified pol δ4 (upper panel) and pol δ3-p12 (lower panel). The amount of the SN BER product was calculated by subtracting the radioactivity of the 16-bp fragment from that of the 25-bp fragment.

Base excision repair (BER) is thought to be a major mechanism in protecting cells from mutagenic base damage spontaneously generated through normal cellular metabolism and also are caused by exogenous agents, such as oxidative stress, hydrolysis, and environmental factors. A typical process of BER is initiated by a group of DNA-*N*-glycosylases and AP endonucleases and mediated by at least two subpathways: single-nucleotide (SN) BER (filling with one nucleotide) and long patch (LP) BER (filling with several nucleotides leading to displacement of the parental strand). Pol β has long been considered to be involved in base excision repair, but other polymerases are likely to play an important role in this pathway as well [33], [40], because a defect in pol β has little impact on cell proliferation [30]. Pol δ, besides its crucial role in DNA replication, also plays a significant role in DNA repair, and is generally regarded as the primary enzyme that performs re-synthesis (gap-filling) in various DNA repair processes [3], [6], [7]. Our previous experiments showed a different behavior between pol δ heterotetramer and trimer lacking p12 to perform translesion synthesis on templates containing base lesions (O^6^-MeG, 8-oxoG, an abasic site or a thyminethymine dimer) [28]. In order to reveal the role of pol δ involved in BER, we examined cell extract and purified protein mediated uracil-intiated BER *in vitro* with uracil-containing plasmid DNA. As shown in Figure 8B, left panel, the pol β −/− cell extract (lane 3, 4) did not show a strong BER deficiency under the conditions in this assay, compared with extract from the wild-type MEF cell line (lane 1, 2). Pol β −/− cell extract still retained significant repair activity, suggesting the presence of a pol β-independent BER back-up pathway. We depleted pol δ four-subunit complex from wild type MEF and pol β −/− cell extract by using the antibodies against its individual four subunits (Figure 8B, right panel). As expected, the level of BER significantly decreased, but a substantial level of BER still persisted in pol δ–depleted pol β −/− cell extract (lane 7, 8), which implies that pol δ plays a significant role in BER. On the other hand, there may also exist other possible pol β-independent BER pathways, for example, pol λ or pol q.

To understand how pol δ plays a role in BER, we monitored the ratio of two BER subpathways in reconstitution BER assay with purified recombinant proteins by measuring the SN BER and LP BER repair patch size using uracil-containing plasmid pUC19N as described in Materials and Methods. In such case, the *kpn*I-*Xho*I digested repair products resulted in a ^32^P labeled 25-bp fragment in which the uracil was replaced by [^32^P]dCMP at the first C, representing SN BER plus LP BER, whereas incorporation of [^32^P]dCMP at the second C and beyond resulted in a ^32^P labeled 16-bp fragment, representing LP BER (Figure 8A). The amount of the SN BER product was calculated by subtracting the radioactivity of the 16-bp fragment from that of the 25-bp fragment. As shown in Figure 8C and 8D, pol δ4 is more likely to mediate LP BER (58%), compared with 42% of SN BER, while pol δ3-p12 is more likely to mediate SN BER (66%), compared with 34% of LP BER. Thus, it seems likely that loss of p12 modulates the rate of SN BER and LP BER during the repair process. These findings provide novel insights into the role of p12 in human pol δ function and into the potential cellular consequences of the *in vivo* conversion of pol δ4 to pol δ3-p12 [27]. It should be noted that in our experimental condition, there are a lot of intermediate products between 41-bp and 25-bp, also below 25-bp, especially in cell extract-mediated BER reaction (Figure 8B), which is probably due to some nicks in the plasmid DNA. Also, we did not see much difference in LP and SN BER products between wild-type MEF and pol β −/− cell extract. This could be due to the incubating reactions too long and the substrate concentrations are below their *Km*. Much further optimization is needed for our *in vitro* BER assay to avoid these confounding factors.

In summary, our work presents a major advance in the expression and assembling of human DNA pol δ in infected silkworm larvae with a recombinant BmNPV virus in which the cDNAs for pol δ four subunits were constructed into a single virus, each in an individual expression cassette. Highly purified recombinant pol δ, as much as 4 mg heterotetramer from 350 larvae by a standardized protocol, is shown to be functional similar to those from natural sources by rigorous characterization and comparison of its activities and homogeneity. Also pol δ plays a significant role by modulating the rate of SN BER and LP BER during the repair process through its conversion from heterotetrameric to trimeric form lacking p12. Pol δ is a key enzyme in DNA replication and repair, and defects in pol δ have been linked to genomic instability. Our research provides a simple novel method for the large scale preparation of human DNA pol δ, which makes it feasible to study at the biochemical level various cellular processes which may lead to or prevent the development of cancer in human.

## Supporting Information

Figure S1 **Western blotting analysis for the generation of recombinant viruses for pol δ subassemblies.** A set of recombinant viruses for pol δ subassemblies were prepared by the transfection of BmN cells with the corresponding recombinant BmNPV bacmid DNAs. The infected cell pellets were run on 12.5% SDS-PAGE and Western-blotted with the indicated antibodies. M: the positions in kDa of protein markers. Lane 1–2: catalytic subunit p125 alone. Lane 3–4: dimer enzyme pol δ-core. Lane 5–6: trimer pol δ-p12 lacking p12. Lane 7–8: trimer pol δ-p68 lacking p68. Lane 9–10: heterotetramer complex. Pol δ four subunits are marked by arrows.
(TIF)

Figure S2 **Purification of recombinant pol δ heterotetramer by Mono Q chromatography from pellet fraction of hemolymph.** The peak fractions from the immunoaffinity chromatography step in which the column was applied with pellet fraction of infected larvae hemolymph were combined and passed through a Mono Q column. The lysates (BC) and the eluted fractions were analyzed by 12.5% SDS-PAGE followed by Coomassie Blue staining. Pol δ four subunits are marked by arrows.
(TIF)
