# Supplementary figures and images for: Production of Recombinant Human DNA Polymerase Delta in a Bombyx mori Bioreactor
Source: PLoS One. 2011 Jul 15;6(7):e22224. doi: 10.1371/journal.pone.0022224 (PMC3137619; doi:10.1371/journal.pone.0022224)

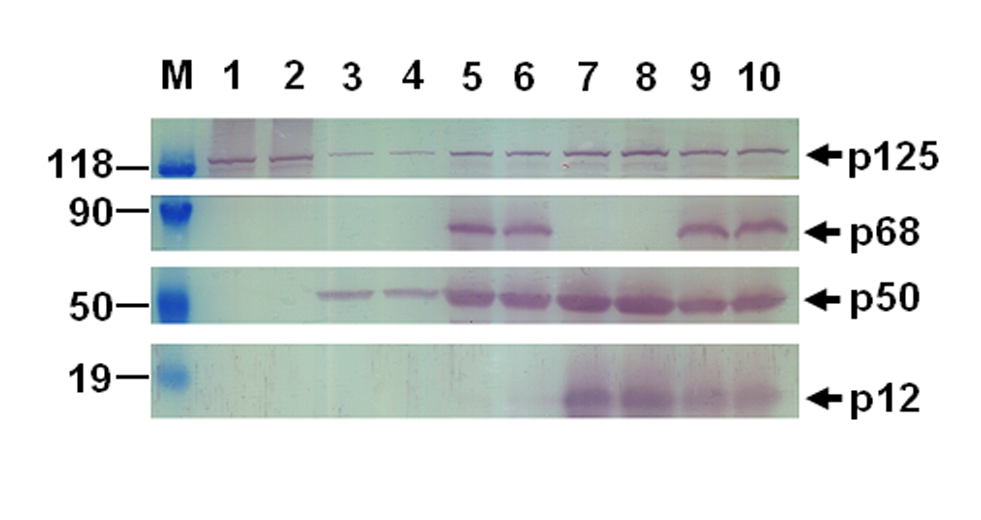

Supplement: Figure S1 — Western blotting analysis for the generation of recombinant viruses for pol δ subassemblies. A set of recombinant viruses for pol δ subassemblies were prepared by the transfection of BmN cells with the corresponding recombinant BmNPV bacmid DNAs. The infected cell pellets were run on 12.5% SDS-PAGE and Western-blotted with the indicated antibodies. M: the positions in kDa of protein markers. Lane 1–2: catalytic subunit p125 alone. Lane 3–4: dimer enzyme pol δ-core. Lane 5–6: trimer pol δ-p12 lacking p12. Lane 7–8: trimer pol δ-p68 lacking p68. Lane 9–10: heterotetramer complex. Pol δ four subunits are marked by arrows. (TIF) [file pone.0022224.s001.tif]

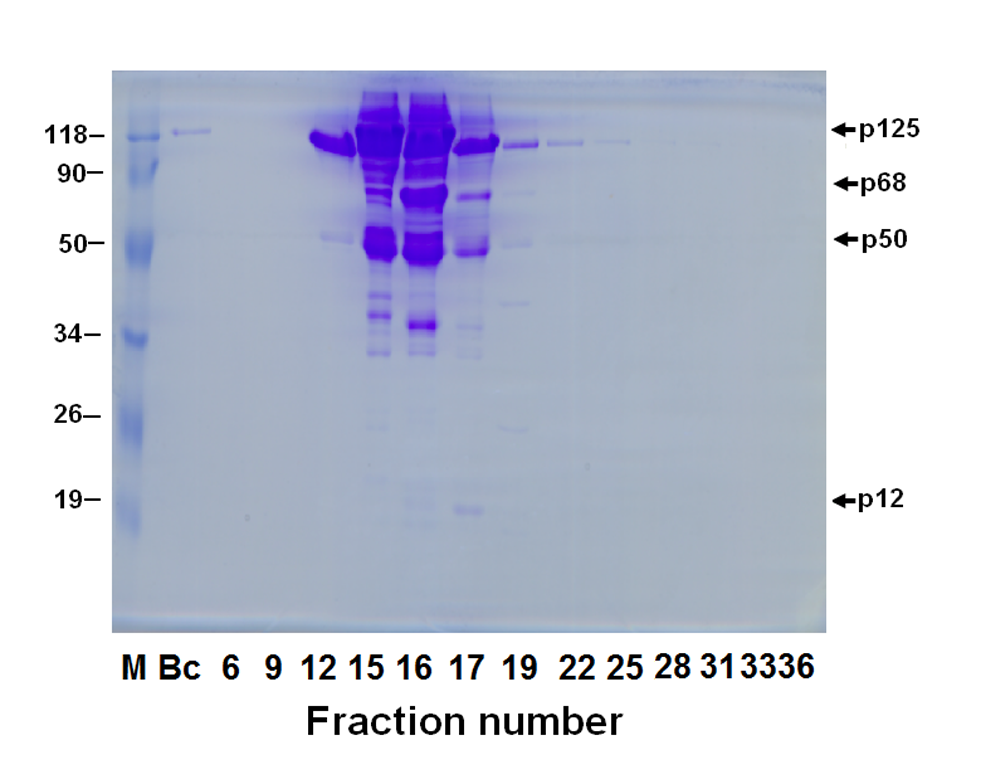

Supplement: Figure S2 — Purification of recombinant pol δ heterotetramer by Mono Q chromatography from pellet fraction of hemolymph. The peak fractions from the immunoaffinity chromatography step in which the column was applied with pellet fraction of infected larvae hemolymph were combined and passed through a Mono Q column. The lysates (BC) and the eluted fractions were analyzed by 12.5% SDS-PAGE followed by Coomassie Blue staining. Pol δ four subunits are marked by arrows. (TIF) [file pone.0022224.s002.tif]
